# Application of artificial intelligence in the analysis of asbestos fibers

**DOI:** 10.3389/fpubh.2025.1584136

**Published:** 2025-07-08

**Authors:** Richard Lee, Drew Van Orden, Suzanne Blanda, John Mihalick, David Bickford, Patrick Metsch

**Affiliations:** ^1^RJ Lee Group, Inc., Pittsburgh, PA, United States; ^2^Consultant, Trafford, PA, United States

**Keywords:** asbestos, automation, identification, artificial intelligence, amphibole

## Abstract

Automated asbestos fiber detection and identification has been the goal of asbestos microscopists for decades. The advent of inexpensive memory, fast digital processing, machine learning, and microscope automation provide the enabling platform for success. This paper will review recent developments in fiber detection and identification by PCM and SEM and will present recent progress in employing artificial intelligence in the TEM classification of asbestos and non-asbestos amphiboles in the evaluation of elongated minerals in raw materials. To date, this project has been self-funded.

## 1 Background

Background and rationale: two topics have been the subject of numerous meetings and publications: namely, what properties should define an amphibole asbestos fiber and should non-asbestos amphibole particles be counted as asbestos. Misclassification of non-asbestos particles as asbestos fibers has significant economic and operational impacts, including unnecessary worker controls, costly air monitoring, project delays, and avoidance of valuable resource areas. This misclassification not only affects industry but can also dilute focus on true asbestos hazards.

Not counting asbestos fibers could have significant health implications for exposed populations. Thus, there has been a driving force to develop reliable, robust methods for identification of amphiboles and for the discrimination of asbestos and non-asbestos “fibers” where a fiber is defined as an elongate mineral particle having an aspect ratio of more than a preset value (generally 3:1 or 5:1).

During the review process, it was suggested that the authors provide detail on their background and qualifications to write the first paper on the use of Artificial Intelligence (AI) to discriminate between asbestos and non- asbestos amphibole fibers. Therefore, we have taken the unusual step of providing a short section on our relevant experience and publications.

The authors have been involved in the development of methods for the identification of amphiboles and discrimination between asbestos and non-asbestos amphiboles by Transmission Electron Microscopy (TEM) for more than four decades. Because this paper is, to our knowledge, the first to address the issue of discrimination of asbestos and non-asbestos fibers using AI, we have included a list of relevant papers, as Appendix A, published by the authors and their colleagues. These evolved over time from an emphasis on mineral identification, to evaluation of counting techniques, and then to discrimination between asbestos and non-asbestos amphiboles using rule-based criteria and discriminant analysis. In addition to extensive involvement in research related to the methods for asbestos fiber identification and definition, the authors have been intimately involved in the development and promulgation of standards for TEM asbestos analysis (AHERA, ISO, and ASTM). It is hoped that other “experts” who may become involved in this work would have comparable backgrounds.

Author publications of direct relevance to this paper are included as citations in the referenced articles.

There has been extensive debate about the reliability of TEM asbestos analysis. The original issues with TEM analysis began in the 1970’s during the Reserve mining case (when laboratories simply adopted the 3:1 aspect ratio) used in Phase Contrast Microscopy (PCM) to define fibers. Unfortunately, the scientists at the time did not recognize the implications of the fact that the 3:1 aspect ratio used in the PCM method was regarded as the lower limit on countable structures in environments known to contain asbestos, such as a textile mill (1) and not the defining parameter of an asbestos fiber. As a result, there were no systematic studies on how to discriminate between asbestos and non -asbestos amphibole in the TEM, even though there was an extensive body of mineralogical research.

In the 70’s there was not even a methodology for the identification of potential amphibole asbestos minerals in multimineral non-occupational environments and so a great deal of attention was paid to the development of techniques to identify regulated amphibole minerals in air samples. Over time the identification procedures became well defined, but as pointed out in this paper, the discrimination between asbestos and non-asbestos remained a challenge.

Significance- The impact of this publication is due to the number of potential users of an AI methodology for asbestos fiber identification. There are about 100 TEM laboratories in the US that provide asbestos analysis on a commercial basis. There are another 30–40 academic and governmental laboratories. However, there are thousands of businesses in virtually every aspect of mining and mineral related activity that consume the data provided and 10,000 of workers whose health safety and economic livelihood are impacted by the data. Further, the extension of asbestos analysis to non-commercial mixed dust environments increases the need for robust methods to discriminate between asbestos and non-asbestos mineral particles.

## 2 Introduction

Asbestos fiber analyses have been conducted for decades typically using microscopic techniques but also including macro-techniques such as gravimetry or x-ray powder diffraction. Microscopic techniques, the subject of this paper, require an analyst to examine the sample, locate a possible asbestos fiber, determine if the fiber meets the protocol requirements, and then document the count. Error may occur at any point in this process. For example, observing a possible fiber requires the sample to have appropriate phase transition (for phase contrast microscopy, PCM) or a significant contrast (in scanning electron microscopy SEM and transmission electron microscopy TEM) for the fiber to be visible. Depending on the protocol, the analyst may simply determine if the fiber meets the appropriate size criteria (PCM) or the analyst may perform additional examination of the fiber to determine possible accepted elemental composition or crystallography (SEM and/or TEM) compositions before determining if the fiber meets the counting requirements.

Manual examination of samples, as implied in the processes, can be time-consuming. For this reason, there have been many proposals to automate the examination (2). Lee et al. (3) examined issues related to automating SEM analyses using image analyses. Later, Baron and Shulman (4) developed an automated optical analysis system (Magiscan) which was more successful. Baron produced data comparing manual and automated PCM image analysis. While successful, the Magiscan was not widely adopted. Inoue et al. (5) developed a similar system to the Magiscan that automated PCM analyses for samples of airborne particulate.

Over the last decade there has been considerable work to develop automated techniques for asbestos analysis. One area that has been examined is using artificial intelligence (AI) in these analyses. A review of the literature indicates that there have been about 30 papers published over the last decade, with most of them in the last 5 years seeking to develop AI fiber identification techniques for PCM and SEM. There are several papers on polarized light microscopy using dispersion staining (6, 7), and other papers on optical analyses (8–10). Kuroda (11) recently discussed using florescence microscopy and AI to examine asbestos fibers. At the same meeting, Iida et al. (12) reported using AI to analyze PCM images of simulated samples of amosite and chrysotile.

The development of AI asbestos analyses has been applied to electron microscopy. The work by Biswas and Biswas (13) represents the state of the art in AI driven analysis using deep learning methodology. Using samples of airborne amosite fibers as the training set, they used AI to automate SEM analysis of a sample, using image analysis combined with energy dispersive spectroscopy (EDS) to identify and count fibers in accordance with the specified protocol. Similar procedures were developed by Hiscock et al. (14), Yamamoto et al. (15) for PCM analyses, and Zyuzin et al. (16) for in-situ rock samples.

These prior studies demonstrate that AI can be useful in asbestos analyses. Automatic identification of fibers, however, is not a trivial task. There are many challenges, such as the fact that detecting fibers is fundamentally a two-dimensional problem since the length of fibers is much greater than the width. Pixel resolution becomes a critical problem in locating and identifying mineral fibrils. Ensuring that there is enough contrast to detect the fibers is also a problem. Overlapping particles and cross fibers and bundles are issues that do not seem to have been effectively addressed at this point, though some progress has been made to handle crossed fibers (17).

**Figure 1:**
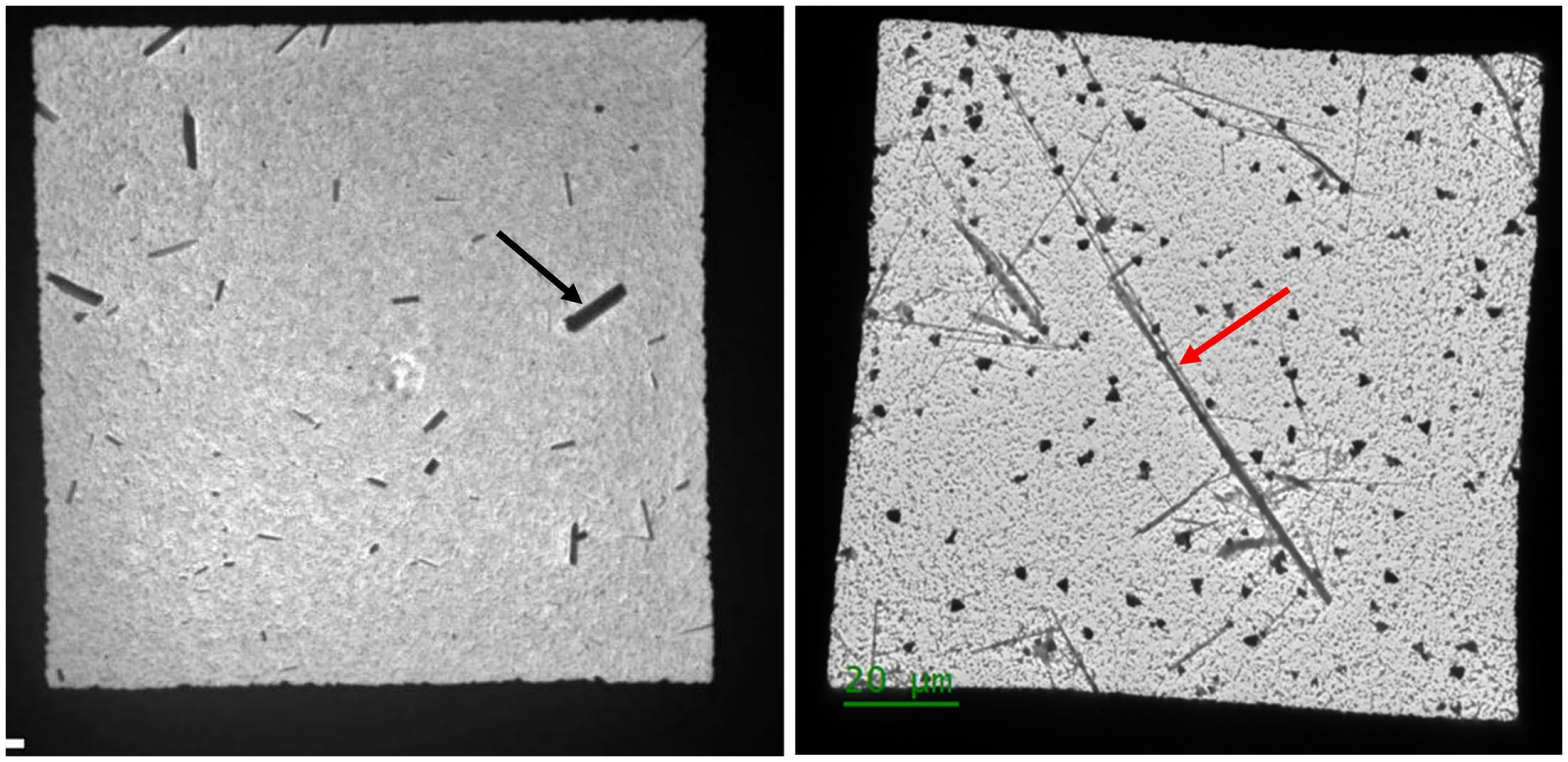
Comparison of images from two different samples as seen in the transmission electron microscope. The left image shows non-asbestos particles (black arrow) while the right shows asbestos particles (red arrow). These morphological classifications originated with an evaluation of the minerals in hand samples and comply with definitions found in accepted protocols (18, 19).

The most important problem is that none of the procedures differentiate between asbestos fibers and non-asbestos particles, an issue of critical importance in the mining and natural mineral environment (Figure 1). The habit of the particles shown in Figure 1 were first determined by examining the bulk minerals (hand-sized) for asbestiform characteristics such as fibrous appearance, easily separable particles, splayed ends, and fibers that, when separated, exhibited tensile strength (that is, could bend without breaking) (18). Such characteristics form the basis for the definition of asbestiform that is incorporated into various analytical protocols (19, 20).

The current methodologies used for air sample analysis/regulation for asbestos fibers rely heavily on traditional optical parameters that were originally developed for PCM. However, these parameters have not been rigorously evaluated or adapted for TEM analyses. This has led to several problems in the field, including: (1) difficulty in distinguishing between asbestos and non-asbestos amphiboles using TEM, as characteristics that are visible in PCM do not translate easily to TEM imaging; (2) widespread use of aspect ratio (length:width) alone to define particles as asbestos fibers, which often leads to over-reporting of fibers that are not asbestos; and (3) ·regulations specifically apply to the asbestos varieties of amphibole minerals due to their adverse health effects, yet many laboratories fail to accurately discriminate between these and non-asbestos fibers (21).

Misclassification of non-asbestos particles as asbestos fibers has significant economic and operational impacts, including unnecessary worker controls, costly air monitoring, project delays, and avoidance of valuable resource areas. This misclassification not only affects industry but can also dilute focus on true asbestos hazards.

Van Orden et al. (22) published a flow chart methodology for discriminating between asbestos and non-asbestos minerals (summarized in Appendix B). Prior effort to accomplish the differentiation between asbestos and non-asbestos has been using various discriminant functions. Siegrest and Wylie (23) and Wylie et al. (24) developed early versions of discriminant functions. Later, Chatfield (25) developed a graphical discriminant function. Wylie et al. (26) examined a very large data set compiled from multiple laboratories and determined a different discriminant function. A more recent discrimination process has been linked to a proposed dimensional coefficient of carcinogenicity (27). Each of these functions can be important for the development of an AI process.

Thus, it is imperative that AI identification of asbestos fibers be developed using mineral fibers that have a known morphology (i.e., asbestos or non-asbestos). Both morphologies must be included to properly train the AI algorithm, and not simply rely on a single amphibole data set. As amphibole particles have varying size characteristics (17, 25, 28) where the sizes of asbestos and non-asbestos particles overlap, it is not acceptable to simply use particle dimensions to make such a differentiation. Therefore, any training set must include examples of well characterized particles from both morphologies.

Using AI will reduce bias in the determination of asbestos and non-asbestos and will enhance the productivity of the laboratory. Such determinations are especially important where the asbestos fibers are a small component of an otherwise non-asbestos sample.

## 3 Artificial intelligence and machine learning techniques

Artificial Intelligence utilizing machine learning typically embodies three processes: (1) decision trees; (2) neural or convolutional networks; and (3) deep learning which layers different levels of specificity over one another to arrive at conclusions.

### 3.1 Decision trees

The most basic AI process is the decision tree algorithm. This is a probabilistic algorithm that sets up a series of yes or no answers to arrive at various levels of decision. Such a tree for weather forecasting may start with humidity (is it high or low), moving on to cloud conditions (it is sunny or cloudy), followed by current weather conditions (there is or is not precipitation) to forecast the next day’s weather. In asbestos analyses, decision trees are used in the air methods (such as ISO 10312 and ISO 13794) on the classification of fibers.

In this matter, AI decision trees would be used to determine if a particle’s dimensions meet the counting rules for the protocol in use. For example, if using the AHERA protocol, is the particle longer than 0.5 μm and have an aspect ratio >5:1?

Decision trees would also be used to evaluate the morphology of a fiber. Are the sides parallel, are the ends perpendicular, and is the surface of the fiber smooth could be aspects of the evaluation.

Concurrently with the decision trees, AI will involve neural networks.

### 3.2 Neural networks

A neural network is a probabilistic algorithm which may be used to evaluate various aspects of mineral or fiber identification. As part of a TEM analysis, the elemental composition (EDS) of the particle is collected. The Neural network would examine the spectrum to determine the elemental composition and compare it to known standards to identify the mineral. The network would also examine the diffraction pattern (typically a selected area electron diffraction pattern, SAED) to establish the particle’s crystallographic dimensions. Combining EDS and SAED will lead to tentative mineral identification.

### 3.3 Deep learning

Deep learning combines the above processes to determine the identification of the mineral and whether the mineral is asbestos or non-asbestos. Depending on the outcome, AI will assign the classification, determine if it should be counted, and should also provide a statement of confidence on the decision. Ultimately, the microscopist makes the final decision. Such decisions are made on a particle-by-particle basis. It is only on such a basis that determination of a small asbestos component in an otherwise non-asbestos sample is possible.

## 4 Training sets

Critical to the development of a successful AI system are the training sets used to develop algorithms. The sets need to be comprised of samples that are defined as either “asbestos” or “non-asbestos” when viewed in hand samples. The sets should avoid samples that comprise both forms (asbestos and non-asbestos) of the mineral. To begin, the samples should also be limited to the regulated minerals (riebeckite asbestos, grunerite asbestos, tremolite asbestos, actinolite asbestos, anthophyllite asbestos, and chrysotile); other minerals of interest such as winchite and richterite can be added later.

A preliminary set of mineral samples is available as shown in Table 1. These samples are either widely acknowledged as occurring in one habit (such as the HSE standards and Jamestown tremolite asbestos) or they clearly exhibit characteristics in a hand sample of one or the other morphology. Additional samples will be obtained to supplement those in Table 1.

**Table 1:** Select reference mineral standards.

Sample	Mineral	Hand sample habit
Jamestown	Tremolite	Asbestos
North Carolina	Tremolite	Asbestos
Shinness	Tremolite	Non-asbestos
South Africa	Riebeckite	Asbestos
Wittenoon	Riebeckite	Asbestos
HSE Standard	Tremolite	Asbestos
HSE Standard 2	Actinolite	Asbestos
Penge	Grunerite	Asbestos
Brockman	Riebeckite	Non-asbestos
Madagascar	Tremolite	Non-asbestos
West Greenland	Anthophyllite	Non-asbestos

The asbestos samples must appear as polyfilamentous mineral in a hand sample and, when examined in the microscope, should exhibit widths < 0.5 μm and mean aspect ratios 20:1 or greater for fibers 5 μm and longer. The asbestos fibrils will also have parallel sides, orthogonal ends, and show evidence of flexibility (high tensile strength). Asbestos fibrils may also occur as bundles of fiber and can have splayed ends (18). Comparatively, non-asbestos particles will have lower aspect ratios, be generally wider than asbestos fibers, and may have varying morphology (parallel sides, non-parallel sides, stepped sides, etc.). These dimensional characteristics have previously been documented (17, 25, 28).

While it is of importance for the correct analysis of all asbestos minerals (both chrysotile and amphiboles) to contemporary industry, the focus of the initial training sets should be on the amphibole minerals due to the difficulty in determining the asbestos/non-asbestos classification in these minerals.

### 4.1 Creation and analysis of training sets

Each sample in the training should be processed in as similar a manner as possible. Each sample is pulverized, suspended in deionized water using an ultrasonic transducer, and an aliquot of suspension deposited onto a polycarbonate filter (0.2 μm pore size). The filter is then prepared for TEM analysis (17). Care should be taken if a settling procedure is used (25) as this may minimize larger particles (especially in width) from some non-asbestos samples.

During the analysis, at least 100 fibers (or particles) longer than 5 μm are examined as these are most closely associated with disease causation (26). For each fiber observed, a photograph of the overall fiber is taken (with magnification adjusted so the fiber fills the photograph image); a photograph of each end (with the magnification increased to show the end clearly) is also taken. Depending on the overall length of the fiber, it may be advantageous to take a photograph of the middle portion of the fiber (again, with the microscope magnification increased). It is possible in asbestos samples that a selected fiber is a bundle of fibers. The ultrasonic processing of the sample should minimize this occurrence but will not eliminate it. However, since bundles occur in nature, it may be appropriate to include these in the analysis.

**Figure 2:**
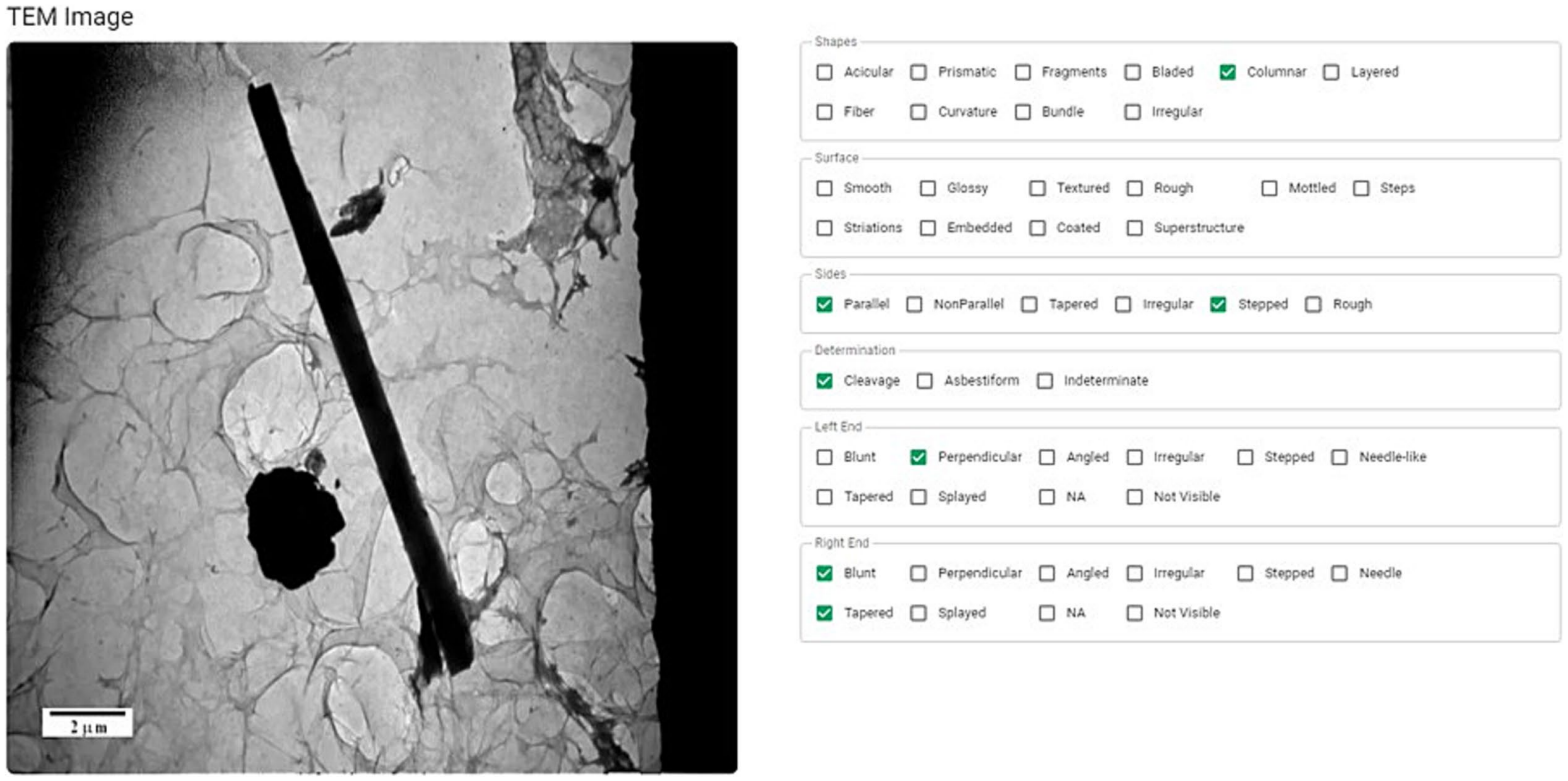
The morphology of each particle (shape, surface, sides, and ends) is examined and characterized using accepted terminology (27).

The data (dimensions, EDS, SAED, and photos) are uploaded to a database for evaluation. Within the database, all images for each fiber are examined, and visible characteristics (shape, surface, sides, and ends) are identified as shown in Figure 2. These morphological characteristics have previously been defined (29) and should be familiar to the expert reviewing the images. To minimize bias in these characterizations, multiple experts (preferably not from the same organization) should evaluate each fiber to reach a consensus description.

In a similar manner, both the EDS and SAED patterns are examined, and the mineral is identified (riebeckite, tremolite, etc.).

**Figure 3:**
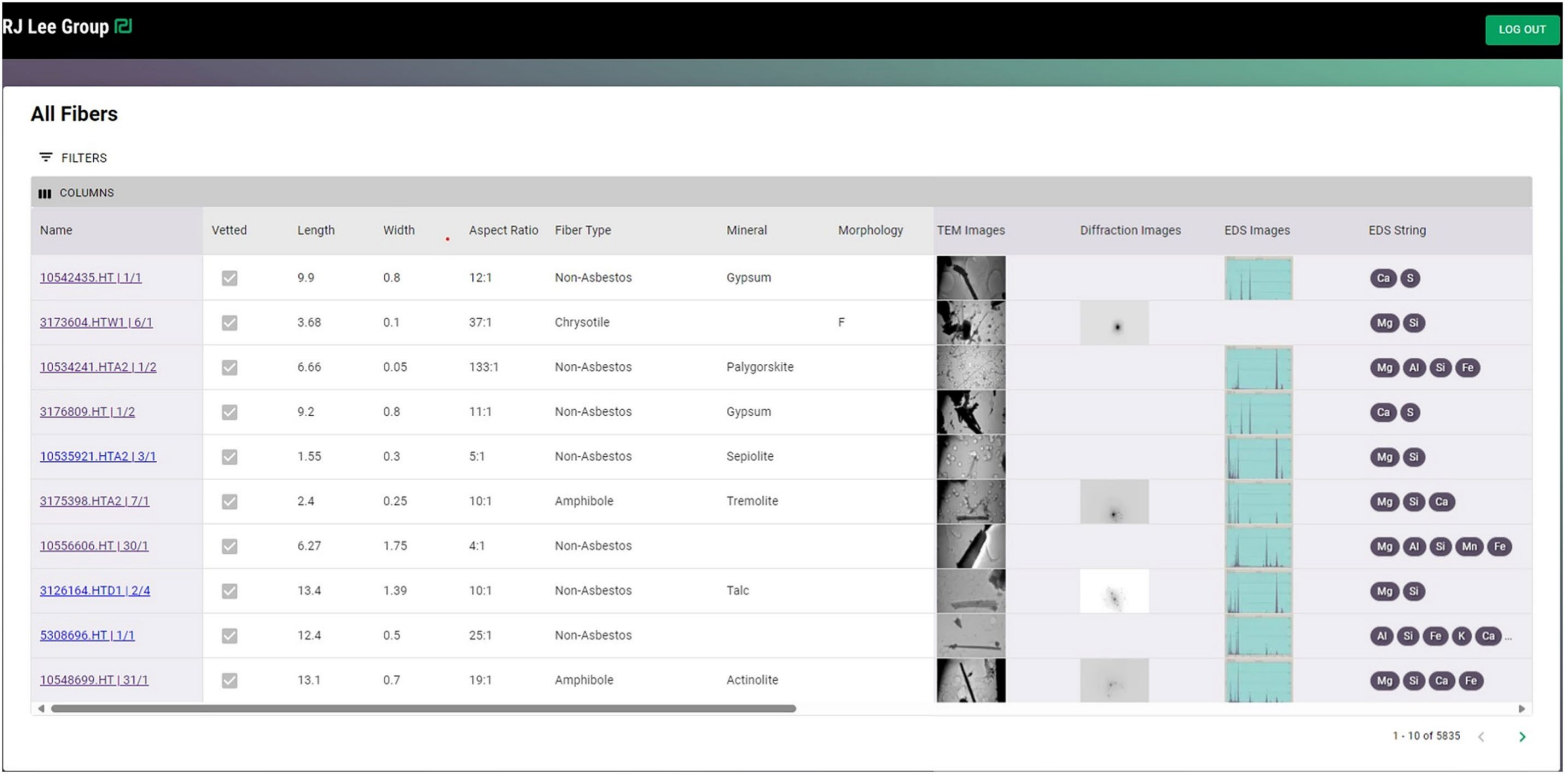
An example of data uploaded to a database showing the image, diffraction pattern, and elemental composition of each particle.

As the fiber analyses are completed, the data can be uploaded to the database (Figure 3), which can be used to train the AI.

## 5 Methods of applied artificial intelligence

The accurate classification of fibers as asbestos or non-asbestos is critical in industrial hygiene, environmental monitoring, and public health to mitigate the risks associated with asbestos exposure.

The applied algorithmic method of approach chosen here is decision tree. Decision tree algorithms, a type of supervised machine learning technique, have proven effective in such classification tasks due to their simplicity, interpretability, and ability to handle complex, non-linear data distributions.

### 5.1 Decision tree fundamentals

A decision tree is a flowchart-like structure in which internal nodes represent decision points based on feature values, branches represent outcomes of decisions, and leaf nodes correspond to class labels or predictions. The algorithm recursively partitions the input feature space by selecting the most informative features at each node, as determined by measures such as Gini impurity, information gain, or entropy (30).

Decision tree algorithms are widely used in machine learning due to their simplicity, interpretability, and versatility. Below are the key advantages of using decision tree algorithms:

1. Easy to understand and interpret
  - Decision trees provide a visual and straightforward representation of decision-making processes.
  - Non-technical stakeholders can easily interpret the tree’s structure, which uses simple “if-then” rules.
  - The interpretability of decision trees makes them particularly valuable in fields like healthcare, finance, and legal compliance.
2. Handles both numerical and categorical data
  - Decision trees can handle mixed data types, including continuous numerical variables (e.g., age or income) and categorical variables (e.g., gender or product type).
  - No need to preprocess data extensively (e.g., scaling or encoding) compared to other algorithms like Support Vector Machines (SVMs).
3. Non-parametric nature
  - Decision trees are non-parametric, meaning they do not make assumptions about the underlying data distribution.
  - They work well on datasets that violate assumptions such as linearity or normality.
4. Captures non-linear relationships
  - Decision trees can model complex, non-linear relationships between input features and the target variable.
  - They split the data based on feature interactions, naturally capturing intricate patterns.
5. Robust to missing data
  - Decision trees can handle missing values effectively by splitting on features that are available, without requiring imputation.
  - Some implementations, such as those in scikit-learn, can make predictions even with incomplete feature information.
6. Requires minimal data preparation
  - Decision trees do not require feature scaling (e.g., standardization or normalization) or transformations like PCA.
  - They handle raw data directly, simplifying the preprocessing pipeline.
7. Feature importance
  - Decision trees can rank features by their importance based on their contribution to reducing impurity at splits.
  - This helps in feature selection and understanding which variables are most influential in the model.
8. Flexibility
  - Decision trees can be used for both classification and regression tasks.
  - They adapt well to a variety of domains, including finance, healthcare, and engineering.
9. Scalability
  - Decision trees are computationally efficient for small to medium-sized datasets, making them practical for many real-world applications.
10. Interpretability of rules
  - Decision trees produce explicit rules, which can be codified and used outside the machine learning model (e.g., in expert systems).

### 5.2 Limitations to consider

While decision trees have many advantages, they are prone to overfitting, especially on small datasets, and can be sensitive to small changes in the data (leading to instability). These limitations can be mitigated by using ensemble methods like Random Forests or Gradient Boosted Trees (31, 32).

In summary, decision tree algorithms are favored for their ease of use, ability to handle diverse data types, and transparency, making them a versatile tool in a machine learning practitioner’s toolbox.

### 5.3 Dataset and features

In the context of fiber classification, the dataset includes microscopic or spectroscopic measurements of fiber characteristics, such as dimensions (length, width, and aspect ratio), and elemental composition (via energy dispersive spectroscopy, EDS) and crystallographic characteristics (X-ray diffraction or selected area electron diffraction, SAED). The particles in the dataset are labeled as “asbestos” or “non-asbestos” based on prior expert analysis as described in the above section.

This process is as follows:

1. Data preprocessing: the raw data undergoes preprocessing to handle missing values, normalize numerical features, and encode categorical variables if present. Outlier detection is also employed to minimize the influence of anomalous measurements.
2. Feature selection: relevant features are identified to improve the model’s performance and reduce computational overhead. For our purposes the features chosen for analysis are:
  - Fiber length
  - Fiber width
  - EDS spectrum results
3. Training and testing: the dataset is split into training and testing subsets, often using a stratified split to maintain the class distribution. The decision tree algorithm is trained on the labeled training data, where the model iteratively splits the data based on the feature that provides the highest information gain at each step.
4. Validation and tuning: cross-validation ensures the model’s robustness against overfitting. Hyperparameters such as maximum tree depth, minimum samples per leaf, and splitting criteria are optimized using grid search or random search techniques.
5. Evaluation metrics: model performance is evaluated using metrics such as accuracy, precision, and recall. Given the health implications of misclassification, false negatives (asbestos fibers classified as non-asbestos) are weighted heavily in the analysis and more weight is put on recall metrics rather than pure accuracy.

### 5.4 Advantages and limitations

Decision tree algorithms provide interpretability by producing human-readable rules, making them ideal for regulatory and legal contexts where transparency is paramount. However, they are prone to overfitting on small datasets and may require ensemble methods, such as random forests or gradient boosting, to improve generalizability and handle larger, more complex datasets.

## 6 Application of AI to an example data set

The previously developed discriminant functions and some available training set data have been uploaded into a developing AI system. Using these multiple discriminant functions and the AI training data will provide an estimate of the accuracy of the classification. The test data set comprised approximately 2,500 fibers.

**Figure 4:**
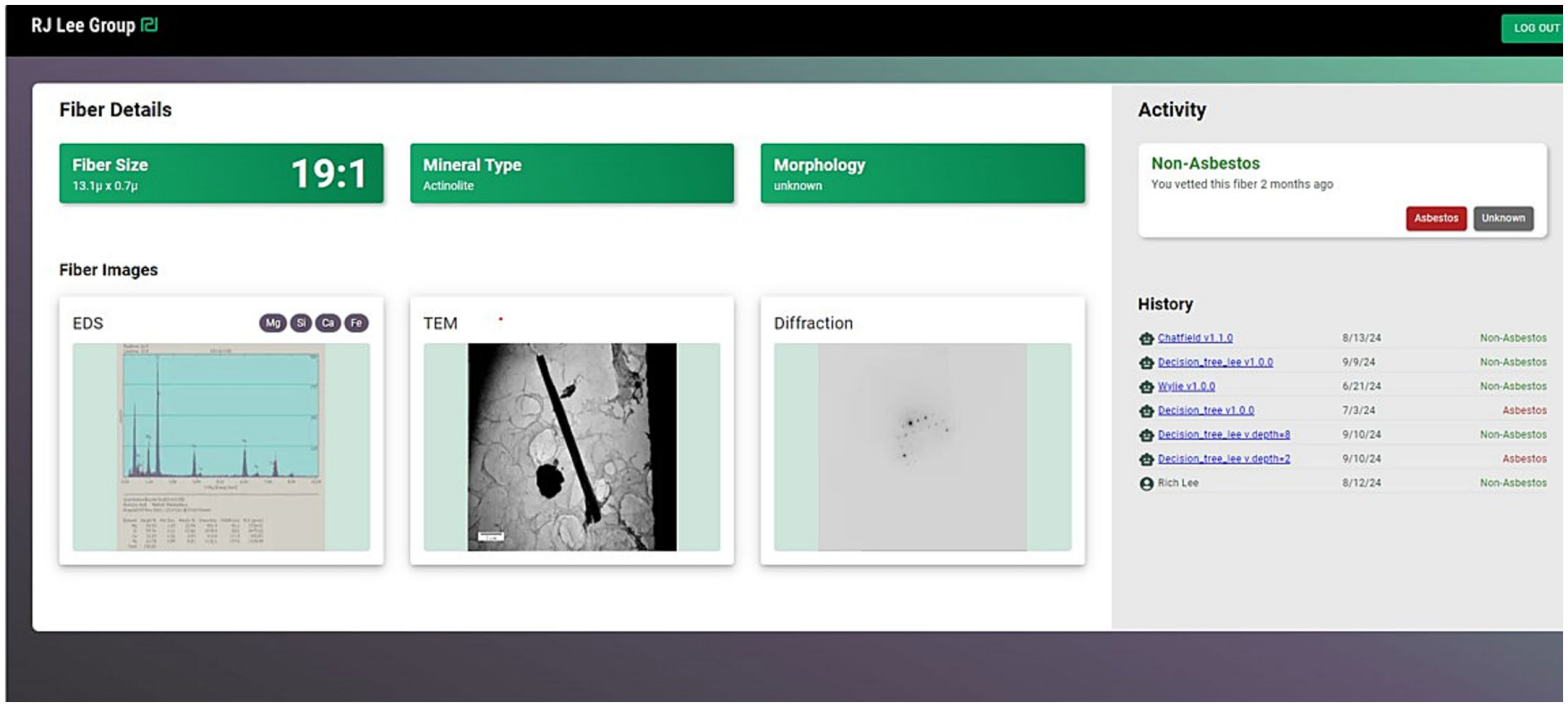
Image showing the AI system application of several discrimination procedures resulting in a consensus classification of the particle as “Non-Asbestos.” The EDS scan, TEM image, and diffraction pattern of the classified particle are shown on the left, while the results of different classification procedures are shown on the right.

As an example, one fiber from a training set was processed through the system as an unknown resulting in a classification of the fiber as “non-asbestos.” As seen in Figure 4, the basic data (EDS, image, and diffraction pattern) are shown along with the results from application of other discrimination procedures (on the right of the Figure). Not all of the discrimination procedures classified the fiber as non-asbestos, with two classifying the fiber as “asbestos.”

**Figure 5:**
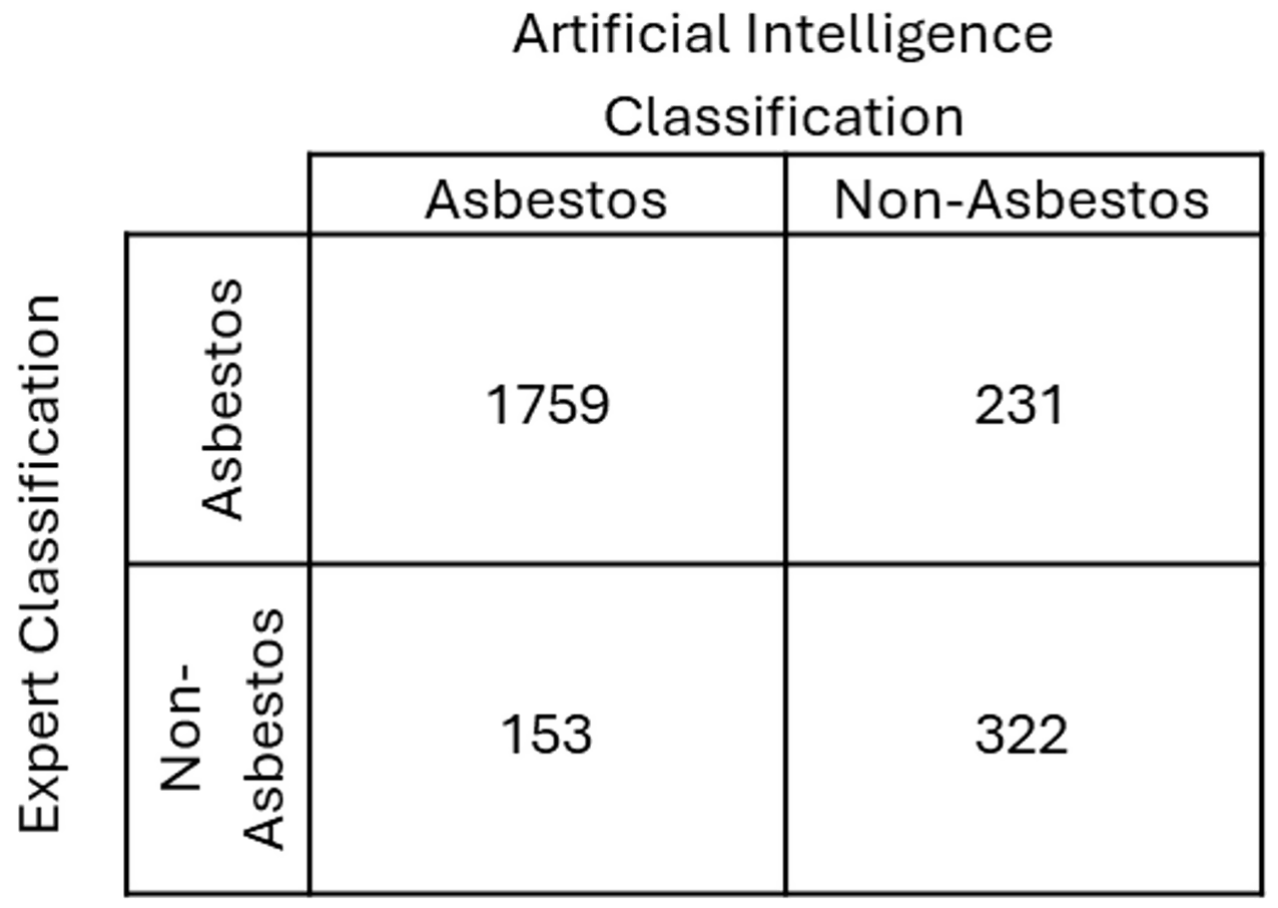
An example of a decision tree (capped at two levels) showing the application of artificial intelligence to an example dataset.

Preliminary results from applying a decision tree classifier to a dataset of fiber samples show high classification accuracy, with significant differentiation between asbestos and non-asbestos fibers based on their physical and chemical properties (Figure 5). Here, the comparison is made between an expert and the AI determinations. Asbestos/Asbestos and Non-Asbestos/Non-Asbestos classifications (expert/AI) show agreement between the two. For this example, 84% of the classifications were consistent between the AI and an expert. The model’s decision rules offer clear insights into the characteristics most predictive of asbestos content, such as high aspect ratios and specific elemental compositions. Where the expert and AI disagree on the classification, additional investigation is needed to determine which characteristics are central to the disagreement,

## 7 Machine learning metrics: accuracy, precision, and recall

When evaluating the performance of a machine learning model, particularly classification models, various metrics provide insights into how well the model predicts the desired outcomes. Below is an explanation of accuracy, precision, and recall, and a discussion on why recall is more appropriate for analyzing asbestos fibers. Here, “True Positive” is defined as both a human expert and the AI classify as particle as “asbestos.” “False Positive” occurs when the AI classifies a particle as “asbestos” and the expert as “non-asbestos.” A “False Negative” occurs when the AI labels the particle as “non-asbestos” and the expert as “asbestos.” Finally, as “True Negative” is when both the expert and AI classify as particle as “non-asbestos.”

Each metric is important, but their relative importance depends on the application: risk analysis, regulatory compliance, or environmental survey.

### 7.1 Accuracy

Definition: accuracy measures the percentage of total predictions that the model got correct. It is defined as:

$$\text{Accuracy}=\frac{\text{True Positives}+\text{True Negatives}}{\text{Total Predictions}}$$

where Total Predictions is the sum of True Positives, True Negative, False Positives, and False Negatives.

Use case: accuracy is a good metric when the classes are balanced (e.g., equal distribution of asbestos and non-asbestos fibers). However, it can be misleading when the dataset is imbalanced.

Example: in a dataset where 95% of the fibers are non-asbestos, a model that predicts “non-asbestos” for every instance would achieve 95% accuracy without actually identifying asbestos fibers.

### 7.2 Precision

Definition: precision measures the proportion of true positive predictions (correctly identified asbestos fibers) out of all instances the model predicted as positive (asbestos fibers). It is defined as:

$$\text{Precision}=\frac{\text{True Positives}}{\text{True Positives}+\text{False Positives}}$$

Use case: precision is important when the cost of false positives is high. For instance, in some scenarios, overestimating the presence of asbestos could lead to unnecessary remediation costs.

Example: if the model predicts 50 fibers as asbestos but only 30 are truly asbestos, the precision is 30/(20 + 30) = 0.6 or 60%.

### 7.3 Recall (sensitivity or true positive rate)

Definition: recall measures the proportion of true positives identified by the model out of all actual positive instances in the dataset. It is defined as:

$$\text{Recall}=\frac{\text{True Positives}}{\text{True Positives}+\text{False Negatives}}$$

Use case: recall is critical when it is more important to identify all actual positive instances, even at the cost of false positives.

Example: if there are 100 actual asbestos fibers and the model identifies 80 of them, recall is 80/(80 + 20) = 0.8 or 80%.

Recall is critical to successful application of AI to asbestos classification for four reasons:

1. Health and safety risks: asbestos fibers are hazardous to human health, potentially causing diseases like mesothelioma and lung cancer. A method which significantly underestimates asbestos fiber concentrations could subject a population to unnecessary risk.
2. Regulatory compliance: environmental and safety regulations often mandate exhaustive detection of asbestos to prevent contamination and health risks. High recall ensures compliance with such standards.
3. Imbalanced dataset: in practice, datasets for fiber analysis are likely imbalanced, with far fewer asbestos fibers compared to non-asbestos fibers. In such cases, accuracy may appear high even if asbestos fibers are frequently missed. Recall directly focuses on identifying all true asbestos fibers.

While accuracy provides a general measure of performance, it may be insufficient in scenarios involving imbalanced datasets. Precision and recall provide deeper insights and are especially useful for unbalanced data sets.

## 8 Approximated, human readable decision tree structure

Decision tree algorithms stand out among machine learning models due to their inherent interpretability and transparency. They are particularly valuable in contexts where human understanding of the decision-making process is as important as the predictions themselves. Decision trees are beneficial for human interpretation for the following reasons:”:

1. Intuitive structure
  - Decision trees are visualized as flowcharts, resembling how humans naturally make decisions: through a series of “if-then” rules.
  - The hierarchical structure, with nodes representing decisions and leaves representing outcomes, makes it easy for non-technical users to follow the logic.
    - Example: if the goal is to classify fibers as asbestos or non-asbestos:
      1. The tree might show a split like: “If a countable fiber width < 0.25 μm, classify as possible asbestos.” Additional branches will be needed to confirm the classification.
      2. This straightforward decision rule mirrors human reasoning and is easy to understand.
2. Transparency and explainability
  - Decision trees clearly articulate why a prediction is made by showing the exact path through the tree that led to the final decision.
  - Unlike black-box models (e.g., neural networks), decision trees reveal the criteria and thresholds used at each step, making them inherently interpretable.
    - Use case: in regulatory or safety-critical industries like asbestos detection, it is vital to explain how a classification was made to ensure compliance and build trust in the model.
3. Identification of key features
  - Decision trees inherently rank features based on their importance, as the algorithm prioritizes the most informative features during splits.
  - This ranking allows users to identify the most influential factors in the decision-making process, aiding domain experts in understanding key variables.
    - Example: a decision tree might show that “parallel sides” is the most critical feature for distinguishing asbestos fibers from non-asbestos particles, helping researchers focus on refining analysis methods for that property.
4. Supports validation and expert feedback
  - Since decision trees align closely with human logic, domain experts can validate the rules and thresholds used in the model.
  - Experts can easily spot unrealistic splits (e.g., thresholds that do not make sense scientifically) and provide feedback to refine the model.

### 8.1 Results of truncated decision tree/aspect ratio key feature

While decision trees are beneficial for interpretation, they can become overly complex or overfit if the data contains noise or too many features. This can reduce their reliability. Techniques like pruning or ensemble methods (e.g., random forests) can balance complexity and accuracy while retaining interpretability.

**Figure 6:**
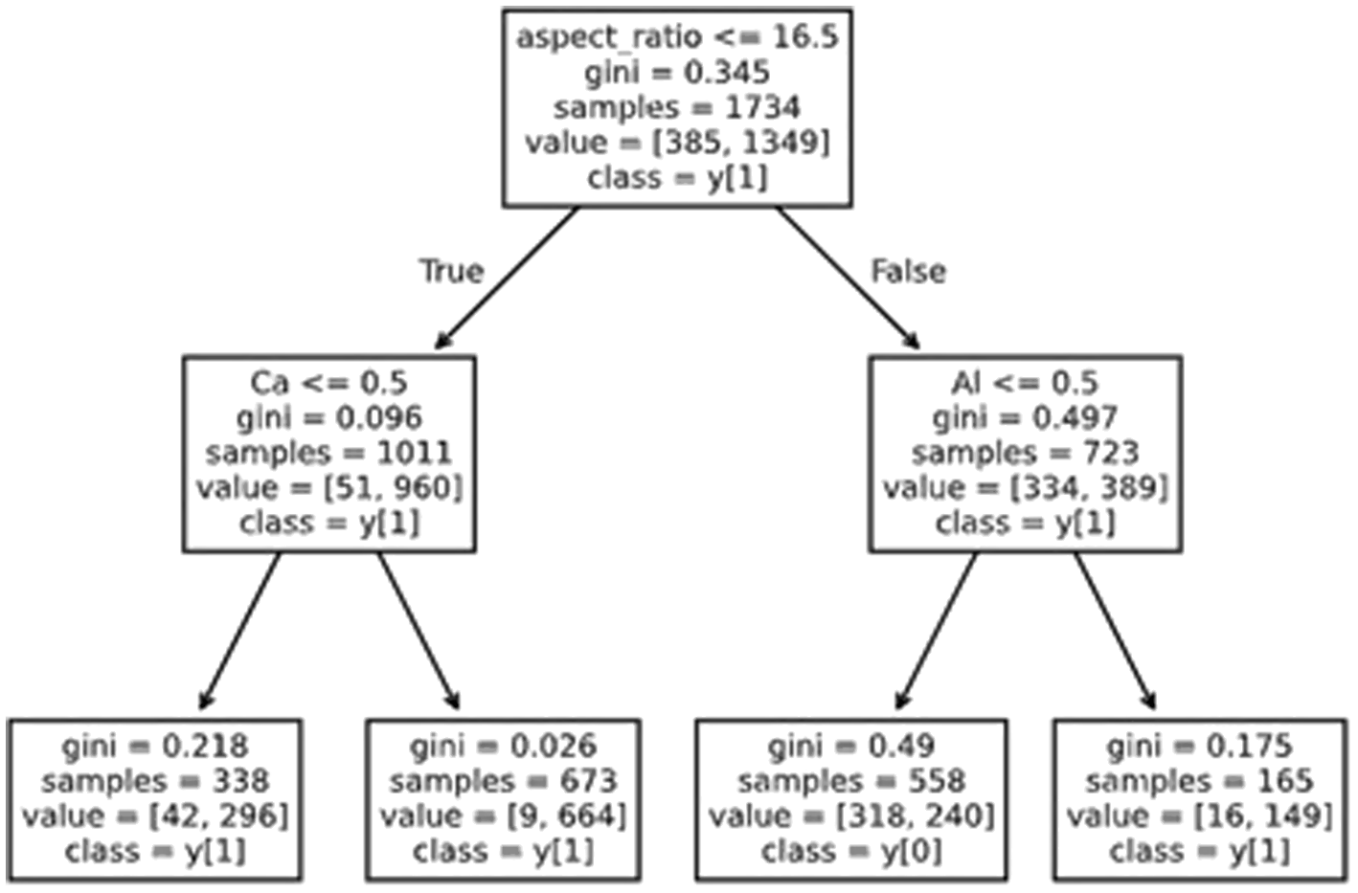
An example of a decision tree (capped at two levels) showing the application of artificial intelligence to an example dataset.

An approach often used is to limit the growth of the tree to a small number of levels/leaves. The resulting decision tree is not as accurate as a fully developed tree (as that can often lead to hundreds of leaves) but is better for human interpretation. A decision tree capped at a maximum depth of 2 levels is illustrated in Figure 6.

Intuitively, this shows that the fiber aspect ratio is a key critical feature. An aspect ratio of <16.5 is the first decision in the tree and therefore the most meaningful in terms of information density.

## 9 Conclusion

The ability of decision trees to produce clear, visual, and logically intuitive decision-making processes makes them uniquely suited for applications requiring human interpretation. In fields like asbestos detection, where safety, compliance, and trust are paramount, the interpretability of decision trees ensures that stakeholders can understand, validate, and act on the model’s predictions.

The preliminary data shows the viability of an AI system, but also points to the need for a larger, more robust set of training samples. The work discussed herein was conducted on mineral samples (hand-sized) that were processed prior to microscopic evaluation. The developed AI procedures are also applicable to samples of airborne or waterborne particles.

These developments are on-going and, upon completion, will be made available to other groups for their use. A set of samples provided by Chatfield are being characterized to expand the reference data set. There is a need for an accepted procedure to validate results so that others may be confident in the use of AI. ISO has established a task group to examine the issues related to asbestos analyses when aided by AI with a suggestion that the group develop a standard to validate AI analyses. Work by other groups on AI asbestos applications continues.

### 9.1 Challenges

Establishing a large, robust training dataset as the definitive source of truth is a complex task. Data is frequently stored across multiple locations in different formats—such as structured and unstructured data, images, PDFs, documents, and spreadsheets—and is difficult to locate due to inconsistent standards and lack of governance (e.g., file naming conventions and storage locations). Additionally, identifying the appropriate data for analysis often requires input from subject matter experts. Vetting and validating the dataset further complicates the process. An important factor when building AI systems is to do so on accurate, reliable data. As such, they can provide valuable insights and support decision-making processes without leading to harmful or misleading expensive outcomes.

## Data Availability

The datasets presented in this article are not readily available because the data reside on proprietary servers. Requests to access the datasets should be directed to R. Lee.

## References

[1] Lynch JR Howard EA. Measurement of asbestos exposure. J Occup Med. (1968) 10:21–4. 5635968

[2] Langer AM. Research perspectives concerning Asbestos minerals and their effects on biological systems. Environ Health Perspect. (1974) 9:335–8. doi: 10.1289/ehp.749335, PMID: 4470954 PMC1475366

[3] Lee R Kelly J Walker J. Automated methods for fiber measurement and identification. VDI-Berichte Nr. (1983) 475:71–89.

[4] Baron BA Shulman SA. Evaluation of the Magiscan image analyzer for asbestos fiber counting. Am Ind Hyg Assoc J. (1987) 48:39–46. doi: 10.1080/15298668791384346

[5] Inoue I Kaga A Yamaguchi K Kamoi S. Development of an automatic system for counting asbestos fibers using image processing. Part Sci Technol. (1999) 16:263–79. doi: 10.1080/02726359808906799

[6] Kawabata K Morishita S Takemura H Hotta K Mishima T Asama H. Development of an automated microscope for supporting qualitative asbestos analysis by dispersion staining. J Robot Mechatron. (2009) 21:186–92. doi: 10.20965/jrm.2009.p0186

[7] Ishizu K Takemura H Kawabata K Asama H Mishima T Mizoguchi H. Automatic counting robot development supporting qualitative asbestos analysis – asbestos, air bubbles, and particles classification using machine learning. J Robot Mechatron. (2010) 22:506–13. doi: 10.20965/jrm.2010.p0506

[8] Cho MO Yoon S Han H Kim JK. Automated counting of airborne Asbestos fibers by a high-throughput microscopy (HTM) method. Sensors. (2011) 11:7231–42. doi: 10.3390/s110707231, PMID: 22164014 PMC3231659

[9] Alexandrov M Ichida E Nishimura T Aoki K Ishida T Hirota R. Development of an automated asbestos counting software based on fluorescence microscopy. Environ Monit Assess. (2015) 187:4166. doi: 10.1007/s10661-014-4166-y, PMID: 25467412

[10] Rabiee A Ventura GD Mirzapour F Malinconico S Bellagamba S Lucci F. Deep learning for asbestos counting. J Hazard Mater. (2023) 455:131590. doi: 10.1016/j.jhazmat.2023.131590, PMID: 37178531

[11] Kuroda A.. (2024). Fluorescent labelling of asbestos fibers. In: *Presented at the Monticello III Conference*, October 22, 2024, Charlottesville, VA.

[12] Iida Y Yamamoto T Iwasaki K Yuki K Nakaji F Yamashiro H Rapid fiber-detection technique by artificial intelligence in microscope images. In: *Presented at the Monticello III Conference*, Charlottesville, VA USA (2024). 10.1093/annweh/wxae014 PMC11033560 38438299

[13] Biswas S Biswas D. Deep learning based asbestos fiber detection. In: *2021 IEEE Applied Imagery Pattern Recognition Workshop (AIPR)*, Washington, DC, USA, 1–5 (2024).

[14] Hiscock M Pisano C Lang C. Machine learning for automated analysis of Asbestos Fibres. Microsc Microanal. (2021) 27:1614–5. doi: 10.1017/S1431927621005948, PMID: 40395388

[15] Yamamoto T Iwasaki K Iida Y Yuki K Kakaji F Yamashiro H. Rapid fiber-detection technique by artificial intelligence in phase-contrast microscope images of simulated atmospheric samples. Ann Work Expo Health. (2024) 68:420–6. doi: 10.1093/annweh/wxae014 38438299 PMC11033560

[16] Zyuzin V Ronkin M Porshnev S Kalmykov A. Automatic asbestos control using deep learning based computer vision system. Appl Sci. (2021) 11:10532. doi: 10.3390/app112210532

[17] Van Orden DR Lee RJ Hefferan CM Schlaegle S Sanchez M. Determination of the size distribution of amphibole asbestos and amphibole non-asbestos mineral particles. Microscope. (2016) 64:13–25.

[18] Ross M Langer A Nord G Nolan R Lee R Van Orden D. The mineral nature of asbestos. Regul Toxicol Pharmacol. (2008) 52:S26–30. doi: 10.1016/j.yrtph.2007.09.008 18423957

[19] Perkins R Harvey B. Method for the determination of asbestos in bulk building materials, EPA/600/R-93/116. EPA Contracts Nos. 68024550 and 68D1000 (1983).

[20] Health and Safety Executive (HSE) (2021). Asbestos: the analysts’ guide, HSG 248.

[21] Lee R Strohmeier B Bunker K Van Orden D. Naturally occurring asbestos: a recurring public policy challenge. J Hazard Mater. (2008) 153:1–21. doi: 10.1016/j.jhazmat.2007.11.079 18180100

[22] Van Orden DR Allison KA Lee RJ. Differentiating amphibole asbestos from non-asbestos in a complex mineral environment. Indoor Built Environ. (2008) 17:58–68. doi: 10.1177/1420326X07087006

[23] Siegrest HG Wylie AG. Characterizing and discriminating the shape of asbestos particles. Environ Res. (1980) 23:348–61. doi: 10.1016/0013-9351(80)90070-5 7202377

[24] Wylie AG Virta RL Russek E. Characterizing and discriminating airborne amphibole cleavage fragments and amosite fibers: implications for the NIOSH method. Am Ind Hyg Assoc J. (1985) 46:197–201. doi: 10.1080/15298668591394653

[25] Chatfield EJ. Measurement of elongate mineral particles: what we should measure and how do we do it? Toxicol Appl Pharmacol. (2018) 361:36–46. doi: 10.1016/j.taap.2018.08.010, PMID: 30134140

[26] Wylie AG Korchevskiy AA Van Orden DR Chatfield EJ. Discriminant analysis of asbestiform and non-asbestiform amphibole particles and its implications for toxicological studies. Comput Toxicol. (2022) 23:100233. doi: 10.1016/j.comtox.2022.100233

[27] Korchevskiy AA Wylie AG. Habit of elongate amphibole particles as a predictor of mesothelial carcinogenicity. Toxicol Rep. (2025) 14:101908. doi: 10.1016/j.toxrep.2025.101908, PMID: 39911320 PMC11795147

[28] Van Orden DR Lee RJ Allison KA Addison J. Width distributions of asbestos and non-asbestos amphibole minerals. Indoor Built Environ. (2009) 18:531–40. doi: 10.1177/1420326X09341503

[29] Campbel W Blake R Brown L Cather E Sjoberg J. Selected silicate minerals and their asbestiform varieties. Washington, DC: US Department of the Interior, Bureau of Mines, IC8751 (1977).

[30] Quinlan J. Induction of decision trees. Mach Learn. (1986) 1:81–106. doi: 10.1023/A:1022643204877

[31] Breiman L. Bagging predictors. Mach Learn. (1996) 24:123–40. doi: 10.1023/A:1018054314350

[32] Breiman L. Random forests. Mach Learn. (2001) 45:5–32. doi: 10.1023/A:1010933404324

