# Supplementary material for: Application of artificial intelligence in the analysis of asbestos fibers
Source: Front Public Health. 2025 Jul 8;13:1584136. doi: 10.3389/fpubh.2025.1584136 (PMC12279743; doi:10.3389/fpubh.2025.1584136)
Supplement: Supplementary file 1 [file Supplementary_file_1.docx]

**Appendix A: Chronological list of author publications on amphibole asbestos identification and counting**

Lally, J.S., R.J. Lee, "Computer Indexing of Electron Diffraction Patterns Including the Effect of Lattice Symmetry,” Proceedings Annual Meeting of the Electron Microscopy Society of America, Boston, MA, 1977.

Lee, R.J., "Electron Optical Identification of Particulates,” Presented at the Symposium on Electron Microscopy of Microfibers, University Park, PA, August 1976.

Lally, J.S., R.J. Lee, "Computer Indexing of Electron Diffraction Patterns Including the Effect of Lattice Symmetry,” Proceedings Annual Meeting of the Electron Microscopy Society of America, Boston, MA, 1977.

Lee, R.J., "Basic Concepts of Electron Diffraction and Asbestos Identification Using SAD. Part I: Current Methods of Asbestos Identification Using SAD,” Scanning Electron Microscopy, Vol. 1, SEM Inc., AMF O'Hare, IL, 1978.

Lee, R.J., J.S. Lally, R.M. Fisher, "Important Considerations in the Identification and Counting of Mineral Fragments,” Presented at the Workshop on Asbestos: Definitions and Measurement Methods, Gaithersburg, MD, July 1977.

Lee, R.J., J.S. Lally, R.M. Fisher, "Identification and Counting of Mineral Fragments,” Proceedings of the Workshop on Asbestos, U.S. Department of Commerce, pp 387-402, 1978.

Lee, R.J., R.M. Fisher, "Identification of Fibrous and Non-Fibrous Amphiboles in the Electron Microscope,” Proceedings International Conference on The Scientific Basis for the Public Control of Environmental Health Hazards, New York, NY, June 1978.

Lee, R.J., "Automated Characterization of Fibrous and Nonfibrous Minerals,” Proceedings of the Denver Research Institute, Aspen, CO, September 1979.

Lee, R.J., R.M. Fisher, "Identification of Fibrous and Nonfibrous Amphiboles in the Electron Microscope,” Annals of the New York Academy of Science, Vol. 330, pp 645-660, December 1979.

Fisher, R.M., A. Szirmae, R.J. Lee, J.L. Hutchinson, "Electron Microscopy of Amphibole Asbestos Fibers,” Proceedings of the Electron Microscopy Society of America, 1980.

Lee, R.J., J.F. Kelly, J.S. Walker, "Considerations in the Analysis and Definition of Asbestos Using Electron Microscopy,” Proceedings NBS/EPA Asbestos Standards Workshop, Gaithersburg, MD, October 1980.

Fisher, R.M., R.J. Lee, J.J. McCarthy, "Applications of Computers in Electron Microscopy,” Proceedings of the Electron Microscopy Society of America Annual Meeting, Clairton Publishing Division, March 1982, Ultramicroscopy, Vol. 8, pp 351-360, 1982.

Lee, R.J., I.M. Stewart, "Living with TEM Clearance,” ECON, April 1988.

Corn, M., K. Crump, D.B. Farrar, R.J. Lee, D.R. McFee, "Airborne Concentrations of Asbestos in 71 School Buildings," Regulatory Toxicology and Pharmacology, Vol. 13, pp 99-114, March 1991.

Lee, R.J., D.R. Van Orden, M. Corn, K.S. Crump, "Exposure to Airborne Asbestos in Buildings," Regulatory Toxicology and Pharmacology, Vol. 16, pp 93-107, March 1992.

Wylie, A.G., K.F. Bailey, J.W. Kelse, R.J. Lee, "The Importance of Width in Asbestos Fiber Carcinogenicity and its Implications for Public Policy,” American Industrial Hygiene Association Journal, Vol. 54, Number 5, pp 239-252, May 1993.

Van Orden, D.R., R.J. Lee, K.M. Bishop, D. Kahane, R. Morse, "Evaluation of Ambient Asbestos Concentrations in Buildings Following the Loma Prieta Earthquake,” Regulatory Toxicology and Pharmacology, Vol. 21, pp 117-121, June 1994.

Oehlert, G.W., R.J. Lee, D.R. Van Orden, "Statistical Analysis of Asbestos Fiber Counts,” Environmetrics, Vol. 6, pp 115-126, 1995.

Lee, R.J., T.V. Dagenhart, G.R. Dunmyre, I.M. Stewart, D.R. Van Orden, "Effect of Indirect Sample Preparation Procedures on the Apparent Concentration of Asbestos in Settled Dusts,” Environmental Science & Technology, Vol. 29, No. 7, pp 1728-1736, 1995.

Lange, J.H., K.W. Thomulka, R.J. Lee, G.R. Dunmyre, "Evaluation of Lift and Passive Sampling Methods During Asbestos Abatement Activities,” Bulletin of Environmental Contamination and Toxicololgy, Vol. 55, No. 3, pp 325-331, 1995.

Lee, R.J., T.V. Dagenhart, G.R. Dunmyre, I.M. Stewart, D.R. Van Orden, "Response to Comment on 'Effect of Indirect Sample Preparation Procedures on the Apparent Concentration of Asbestos in Settled Dusts',” Environmental Science & Technology, Vol. 30, No. 4, pp 1405-1406, 1996.

Lee, R.J., D.R. Van Orden, G.R. Dunmyre, "Interlaboratory Evaluation of the Breakup of Asbestos-Containing Dust Particles by Ultrasonic Agitation,” Environmental Science & Technology, Vol. 30, Number 10, pages 3010-3015, 1996.

Lee, R.J., D. Van Orden, I.M. Stewart, "Dust and Airborne Concentrations - Is there a Correlation?”, Advances in Environmental Measurement Methods for Asbestos, ASTM Lange, J.H., K.W. Thomulka, R.J. Lee, D.R. Van Orden, "Surface and Passive Monitoring for Asbestos in an Industrial Facility,” Indoor Build Environment, pp 327-333, 2002.

S Lee, R.J., D.R. Van Orden, W.H. Powers, K.A. Allison, "Implications of Analytical Techniques for Asbestos Identification,” Presented at the National Stone, Sand & Gravel Association's Environment, Safety and Health Forum, September 2001.

Bailey, K.F., J. Kelse, A.G. Wylie, R.J. Lee, "The Asbestiform and Nonasbestiform Mineral Growth Habit and Their Relationship to Cancer Studies,” A Pictorial Presentation, 2003.

Van Orden, D.R., R.J. Lee, S.R. Badger, "Characterizing Asbestos Fiber Comminution Resulting from Preparation of Environmental Samples," Powder Technology, Vol 162, pp 183-189, 2006.

Harris, K. E., K. L. Bunker, B. R. Strohmeier, R. Hoch, and R. J. Lee, “Discovering the True Morphology of Amphibole Minerals: Complementary TEM and FESEM Characterization of Particles in Mixed Mineral Dust,” Modern Research and Educational Topics in Microscopy, A. Méndez-Vilas and J. Díaz, Eds., Formatex Microscopy Book Series, No. 3, Vol. 2, Formatex Research Center, Badajoz, Spain, pp. 643-650, 2007.

Strohmeier, B. R., K. L. Bunker, K. E. Harris, R. Hoch, and R. J. Lee, “The Database Solution to Particle-by-Particle Analysis of Mixed Mineral Dust,” Microscopy Today, Vol. 15, No. 6, 44-47, November 2007.

Van Orden, D. R., K. A. Allison, and R. J. Lee, "Differentiating Amphibole Asbestos from Non-Asbestos in a Complex Mineral Environment," Indoor and Built Environment, Vol. 17, pp. 58-68, 2008.

Lee, R. J., D. R. Van Orden, "Airborne Asbestos in Buildings", Regulatory Toxicology and Pharmacology, Vol. 50, Issue 2, pp. 218-225, 2008.

Lee, R. J., B. R. Strohmeier, K. L. Bunker, D. R. Van Orden, “Naturally Occurring Asbestos – A Recurring Public Policy Challenge,” Journal of Hazardous Materials, Vol 153, pp.1-21, 2008.

Glenn, R.E., R.J. Lee, L.M. Jastrem, K.L. Bunker, D.R. Van Orden, B.R. Strohmeier, "Asbestos: By Any Other Name, Is It Still?", Occupational Safety and Health Reporter, Vol. 38, No. 22, pp. 428-433, 2008.

Van Orden, D.R., R.J. Lee, M.S. Sanchez, M.D. Zock, "The Size Distribution of Airborne Bolivian Crocidolite Fibers", Annals of Respiratory Medicine, July 2012.

Ilgren, E., D.R. Van Orden, Y. Kamiya, J. Hoskins, "Further Evidence for Fiber Width as a Determinant of Mesothelioma Induction and Threshold--Anthophyllite, Bolivian Crocidolite, and Cape Crocidolite", Annals of Respiratory Medicine, July 2012.

Ilgren, E.B., D. Van Orden, R. Lee, Y. Kamiya, J.A. Hoskins, "Environmental 'Low Dose' Mesotheliomas and Their Relationship to Domestic Exposures-Preliminary Report", Environment and Pollution, Vol. 3, No. 2, pp. 48-54, 2014.

Lee, R.J., D.R. Van Orden, "Letter to the Editor: Asbestos in commercial cosmetic talcum powder as a cause of mesothelioma in women", International Journal of Occupational and Environmental Health, 21, pp. 337-341, 2015.

Ilgren, E.B., D.R. Van Orden, R.J. Lee, Y.M. Kamiya, J.A. Hoskins, "Further Studies of Bolivian Crocidolite–Part IV: Fibre Width, Fibre Drift and their relation to Mesothelioma Induction: Preliminary Findings", Epidemiology Biostatistics and Public Health, Vol. 12, No. 2, 2015, <http://dx.doi.org/10.2427/11167>.

Van Orden, D.R., R.J. Lee, C.M. Hefferan, S. Schlaegle, M. Sanchez, "Determination of the Size Distribution of Amphibole Asbestos and Amphibole Non-Asbestos Mineral Particles", The Microscope, Vol. 64:1, pp. 13-25, 2016.

Lee, R.J., D.R. Van Orden, L.A. Cox, S. Arlauckas, R.J. Kautz, "RE: In response to Harper and Key-Schwartz Letter to the Editor: Preparation of Respirable Crystalline Silica Samples for Subsequent Analysis", Regulatory Toxicology and Pharmacology, Vol. 83, pp, 103, 2017.
