# Supplementary material for: Application of artificial intelligence in the analysis of asbestos fibers
Source: Front Public Health. 2025 Jul 8;13:1584136. doi: 10.3389/fpubh.2025.1584136 (PMC12279743; doi:10.3389/fpubh.2025.1584136)
Supplement: Supplementary file 2 [file Supplementary_file_2.docx]

Appendix B. Van Orden et al (22) Asbestos Classification Rules

1. Does the particle aspect ratio meet the counting criteria? Aspect ratio is >3:1 or >5:1 and length >0.5µm or >5.0µm per the methods, respectively. If not, reject.
2. Does the particle have parallel sides? If not, it is non-asbestos and reject. Parallel sides are essential for forming long, high strength fibers. Much like a beam or piano wire, any variation in width or imperfection becomes a point of weakness and will reduce strength.
3. Does the particle have curvature? A characteristic of asbestos when viewed by optical microscopy is the presence of bundles of curved fibers with splayed ends. Because one is often examining single fibrils in the TEM, the particle may not have splayed ends. Curvature is an indication of flexibility and high strength and is a unique characteristic of asbestos fibers. Curved fibers are checked for identification and if amphibole, generally are asbestos. The exception would be if the Selected Area Electron Diffraction (SAED) pattern shows no signs of twinning or streaking, indicating few defects.
4. Does the particle have perpendicular terminations? Square, right-angle terminations are a characteristic of asbestos amphiboles. They arise because the fineness of the fiber and lack of surface defects suppresses the normal cleavage. Instead, the [100] twinning and Wadsley defects deter [110] crack propagation and provide slip planes and/or grain boundary precipitates/discontinuities for fractures to initiate that are oriented perpendicular to the fiber axis producing perpendicular fractures rather than the tapered, stepped or rough terminations of non-asbestos. In the case of very fine fibers, the ends may appear rounded, but the tangent to the end is still perpendicular. Physically, this can be understood by thinking about breaking a wire along its axis. The force can be dissipated much easier by breaking across the wire.
5. Does the particle exhibit dark contours roughly perpendicular to the fiber axis? Asbestos fibers produce diffraction contours as a result of internal defects and thermal effects. This rule has limited value because the effect is limited to thin particles.
6. Does the diffraction pattern have rows of closely spaced spots, perpendicular to the fiber axis? It is well known that amphibole asbestos is highly twinned and faulted. The natural growth mechanisms create lathe like fibers that tend to orient on their [100] crystal faces. When the electron beam impinges on the particle and a diffraction pattern, rows of parallel spots roughly 5.3A in spacing are produced. RJLG’s process expands this requirement to allow near [100] patterns.
7. Does the diffraction pattern exhibit evidence of twinning or internal defects? Asbestos amphiboles are highly faulted and their SAED patterns exhibit symmetry, forbidden spots, and extension of the spots resembling streaking. If none of these occur, the particle is not likely to be asbestos.
8. Is the pattern consistent with an amphibole? SAED patterns can be measured and compared with theoretical values using computer software. If the pattern is not consistent with an amphibole, it is rejected.
9. Are the X-ray spectra consistent with amphibole asbestos? If not, reject. In modern systems, step 9 is generally performed first.
